# Delineating and preventing plastic waste leakage in the marine and terrestrial environment

**DOI:** 10.1007/s11356-020-08139-y

**Published:** 2020-03-02

**Authors:** John N. Hahladakis

**Affiliations:** Center for Sustainable Development, College of Arts and Sciences, Qatar University, P.O. Box: 2713, Doha, Qatar

**Keywords:** Plastics, Marine litter, Oceans, Plastic waste, Marine pollution, Plastic debris

## Abstract

Plastics are nowadays considered to be the workhorse material of our modern society with an ubiquitous presence that has increased manifold over the past 60 years, providing several benefits to the global economy. However, inappropriate and/or uncontrolled disposal practices, poor waste management infrastructure and application of insufficient recycling technologies, coupled with a lack of public awareness and incentives, have rendered plastic waste omnipresent, littering both the marine and the terrestrial environment with multi-faceted impacts. This short communication/commentary aims at delineating the plastic litter global challenge providing, at the same time, scientific views and perspectives on properly dealing with this material type, both upstream and downstream.

## Introduction

With global drivers and terms such as sustainability, eco-efficiency and dematerialization, the concept of circular economy (CE) is gaining global momentum to the point that a fundamental rethink on plastics, at all the stages of their lifecycle, is required.

There are many definitions or attempts to define CE reported both in scientific and in “grey” literature. It can be characterized, in short, as an “industrial system that is restorative and regenerative by intention and design” (Kaur et al. 2018). Nonetheless, to achieve a successful journey into the “circular” world, it would require the harmonic coexistence and cooperation of all players and stakeholders involved (consumers, decision makers, industries, governmental entities, waste management infrastructure, etc.), plus the development and use of adequate frameworks and tools that will enable us to properly and transparently assess the creation and dissipation of a “multidimensional” value that spans the environmental, social, economic and technical domains (Iacovidou et al. 2017a, b; Millward-Hopkins et al. 2018).

Plastics are nowadays considered to be the workhorse material of our modern society with an ubiquitous presence that has increased manifold over the past 60 years, providing several benefits to the global economy. The European Commission (EC) has recently introduced a European Strategy for Plastics, identifying and setting any action on plastics as a priority in the 2015 CE Action Plan, facts that solidify the significance of this type of material and highlights the need for further future research and investigation (European Commission 2016, 2018). However, their value chain is still treated with the archetypically linear mode of take-make-dispose. It is only during the past few years, together with the launch of the CE concept, in 2010, that the drawbacks of this way of dealing with plastics have been, at last, clearly realized.

Conventional plastics are made of thermoplastic resins and can be generally categorized into seven classes: the polyethylene terephthalate (PET) (known as type 1), high-density polyethylene (HDPE) (known as type 2), polyvinyl chloride (PVC) (known as type 3), low-density polyethylene (LDPE) (known as type 4), polypropylene (PP) (known as type 5), polystyrene (PS) (known as type 6), and others (known as type 7). The latter category refers to multilayer polymer formations, not collected for recycling (Hahladakis and Iacovidou 2018).

In the present short communication/commentary, we will attempt to capture and delineate the main problems, as well as potential solutions, associated with plastic waste that end up as litter in the marine and terrestrial ecosystem, providing, concurrently, generic views and ideas on properly dealing with this material type.

## Plastics: a useful material and a resource to be “handled with care”

Plastics are considered to be one of our modern world’s greatest industrial innovations, applicable to many sectors such as packaging, electrical and electronic equipment (EEE), construction, automotive, etc. Owning to their light weight, durable and multipurpose nature, production of plastics has reached approx. 322 Mt (in 2015), with a projection of doubling this amount by 2035 (Ellen MacArthur Foundation 2016; Geyer et al. 2017; PlasticsEurope 2016). However, inappropriate and/or uncontrolled disposal practices, poor waste management infrastructure and application of insufficient recycling technologies, coupled with a lack of public awareness and incentives, have rendered plastic waste ubiquitous, littering both the marine and the terrestrial environment with multi-faceted impacts (Jambeck et al. 2015, 2018).

Plastics can be mainly classified into three groups, based on their particle size. All plastic materials > 5 mm fall under the category mostly known as “macroplastics” (Axelsson and van Sebille 2017). Nonetheless, due to various conditions and environments (e.g. weather, UV light, seawater), plastics can potentially be degraded and dissociated (fragmented) into smaller pieces, 50–5 mm, called “microplastics” (MPs) (Andrady 2011; Kalogerakis et al. 2017; Wang et al. 2016). Finally, the nanometre-sized plastic particles usually defined in < 100 nm of size constitute the “nanoplastics” group (Koelmans et al. 2015).

It is, in fact, the latter two categories that are considered the most potentially harmful, both to humans and to other living organisms, due to several reasons: (a) after, e.g. entering the marine environment, a large part of it is out of sight sustained in the water column and/or hidden deep inside the benthos, (b) they can easily be ingested or entangled by various species (Browne et al. 2008; Steer et al. 2017; Teuten et al. 2009), and (c) embedded chemical substances are more readily released during the degradation process of these fine particles rather than the larger ones. Furthermore, these tiny pieces that can easily accumulate persistent organic pollutants (POPs) and other substances of concern (SoC) (Chen et al. 2019; Hahladakis et al. 2018b; Koelmans et al. 2013) serve as a pathway to food chain.

### Plastic waste in the marine and terrestrial environment

Estimating the amount of plastic waste or “plastic debris” (as it is also known) leaked into the environment is a quite challenging task, considering (a) the lack of accurate data, (b) the dynamics of the plastics production-generation waste system and (c) the global variations in societal attitudes and in the waste management infrastructure (Dahlbo et al. 2018; Hahladakis and Aljabri 2019; Hahladakis et al. 2018a; Horton et al. 2017). Taking also into account the marine and terrestrial ecosystem interactions (transport mechanisms and pathways), it is difficult to differentiate and distinguish between the plastic particles retained in the aquatic systems and those retained in the terrestrial environments (Horton et al. 2017; Rillig 2012). In addition, since neither particulate material nor solid surfaces are rare in continental systems, this might led to an underestimation and/or a minor scientific attention of plastic waste threats to terrestrial species. Sewage treatment plants, landfills, urban and industrial centres, and continental systems (i.e. terrestrial, aquatic and semiaquatic in land environments), in general, are considered diverse sources of MPs (Lechner and Ramler 2015; Mahon et al. 2017; Talvitie et al. 2017)

The best available estimates, so far, regarding global plastic waste inputs from land-based sources into the oceans come from Jambeck et al. (2015). However, several assumptions were made in this study, and it is considered that the calculations presented therein could perhaps constitute an underestimation of the reality (Horton et al. 2017). Based on different estimations and sources, the oceans could contain more than 150 Mt of plastics (Ocean Conservancy 2015) or more than ca. 5 trillion plastic particles (macro and micro) (Eriksen et al. 2014).

Other researchers have reported that ca. 4900 Mt, out of the estimated 6300 Mt total amount of plastics ever produced, have been disposed either in landfills or inappropriately scattered in the environment. This figure is anticipated to reach ca. 12,000 Mt by 2050, unless immediate actions are taken (Geyer et al. 2017).

**Fig. 1:**
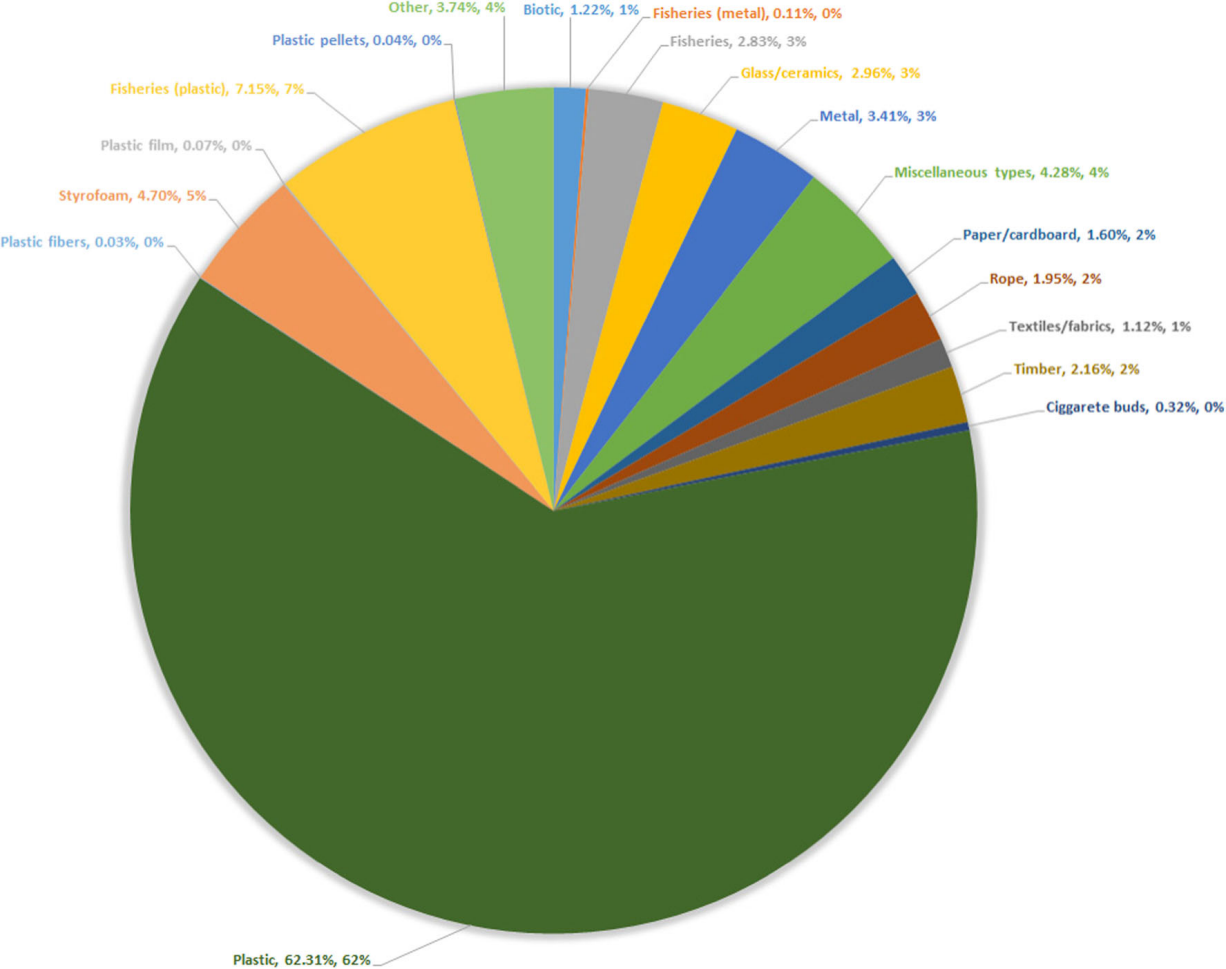
Global composition estimate of marine litter. The percentages of the various types of litter shown in the graph were calculated as the weighted means of all studies under consideration, regardless of units (565 publications, 3982 locations). (Redrawn from source: https://litterbase.awi.de/litter_graph)

According to Awi-Litterbase, a continuously updating database on global marine litter, plastic affiliated debris accounts for approx. 70% of the overall amount of marine litter (Tekman et al. 2019) (see Fig. 1).

There are a number of sources of MPs identified in the environment. Abrasion from car tires and household plastic materials and products, ship paints, discarded products from fishing vessels, aquaculture facilities, merchant ships, recreational boats, offshore oil or gas platforms, as drilling fluids for oil and gas exploration, etc. are just a few to mention. However, exact and reliable estimates on the quantities of the individual sources and/or origins of the different types of plastics are hampered by the complexity of the sources of micro- and macroplastics; the lack of quantitative data on transport and fate in the environment; and the high geographic, hydrographic and geomorphologic variability of the relevance of different sources and introduction routes, which is caused by differences in the infrastructure, especially with regard to waste management (Kalogerakis et al. 2017; Lambert et al. 2014; Law and Thompson 2014).

A detailed study by Schwarz et al. (2019) describes the main sources, transport and accumulation of several types of plastics in various aquatic environments (oceanic, epipelagic, beaches, riverine and freshwaters). In this study, the researchers concluded that PE was proportionally dominant in all environmental compartments, followed by PP and PS. The polymer composition in freshwater, beach, ocean and epipelagic was most homogeneous, and PE, PP and PS together represented on average 92.2% and 95.8% of the encountered polymers, respectively (Schwarz et al. 2019). It is also noteworthy the fact that the vertical movement of plastics in water, or the sedimentation rate, is mainly affected by three parameters: the polymer’s density, its surface area and its particle size (Chubarenko et al. 2016; Kowalski et al. 2016).

Five Asian countries (China, Thailand, Indonesia, the Philippines and Vietnam) and ten rivers (Indus, Ganges, Amur, Hai He, Yellow, Mekong, Pearl, Yangtze, Nile and Niger) are accounted responsible for originating and transporting ca. 90% of the global plastic waste input to the sea (Ocean Conservancy 2015; Schmidt et al. 2017). Relative rough projections report that the amount of plastic litter entering the oceans could triple by 2025, if no interventions are made (Jambeck et al. 2015).

Should the linear “take-make-use-dispose” model of economy continues to prevail and society fails to implement a successful circular economy model, it has been reported that by 2050 plastics present in the oceans will weigh more than fish (Ellen MacArthur Foundation 2016).

It is noteworthy the fact that single-use plastics contribute significantly to this leakage. This is why the UK Government intends to ban all sales of single-use plastics, including plastic straws, from 2019 (Craggs 2018). Single-use plastics (such as bags and food containers) often carry food particles and scents that attract animals, which eat the plastic together with the food. In turn, the plastic blocks the animals’ digestive tracts and, consequently, the passage of food, thereby leading to death by starvation or infection. Birds and large mammals have been found dead after ingesting plastic films and bags. Additionally, birds may use plastic pieces to build their nests; however, newly hatched chicks will peck away at these pieces and potentially swallow them (Craggs 2018).

The main characteristics of plastics (such as nondegradability and persistence) that make them so appealing, popular and useful are, in fact, the main reasons that render plastic waste such an emerging environmental issue. Plastics could stay in the environment for a long period of time; some might take up to centuries to break down, creating various environmental, economic and social impacts; this could harm biodiversity and deplete the ecosystem services and the natural resources needed to support life.

Plastic waste is also considered to be one of the biggest threats to coral reefs by increasing the likelihood of disease outbreaks, thereby threatening marine habitats that provide food, income and cultural benefits to approx. 280 million people (Lamb et al. 2018). Plastic debris stresses coral through light deprivation, toxin release and anoxia, thereby providing pathogens a foothold for invasion (Lamb et al. 2018).

When present in the marine and aquatic environments, plastics are fragmented into smaller pieces (micro- and nanoplastics) (Andrady 2011; Koelmans et al. 2015) which threaten marine biodiversity (Browne et al. 2013). The initial breakdown of a polymer is dependent on various physical and biological forces. Biodegradation is defined by several steps that could be identified by specific terminology. The first step is the biodeterioration, the second step is the depolymerization, and, finally, we have the assimilation and mineralisation (Lucas et al. 2008). The generation of MPs via the fragmentation of larger plastic particles is a complex and multifactorial process, depending on various parameters, such as luminance, temperature, and oxygen level, and characteristics inherent to the nature of the degrading material, e.g. its molecular weight distribution and presence of additives (Hahladakis et al. 2018b; Kalogerakis et al. 2017). Environments where plastics are exposed to mechanical stress or high oxidizing radiation will accelerate their deterioration; such environments may be a substantial source of MPs.

Inevitably, MPs will enter the food chain, and being, also, able to accumulate high concentrations of POPs and other SoC (Hahladakis et al. 2018b), it will inevitably serve as a pathway for their transfer to aquatic and marine organisms (Rochman et al. 2013) and consequently human health (Worm et al. 2017). It is worth mentioning that there have been several attempts and calls for MPs to be regarded as POPs, owning to their pervasive and persistent nature (Hurley et al. 2018). Nonetheless, there is currently no scientific proof and/or evidence that they pose direct harm to human health.

Other researchers report that MPs constitute, also, an emerging source of soil pollution (Rillig 2012). The impacts of MPs in several media and ecosystems (e.g. soils, sediments, freshwater) could potentially be proved long term and damaging, through adverse effects on organisms, i.e. soil-dwelling invertebrates and fungi (Souza Machado et al. 2018). A significant number of MP particles have been found in organic fertilizers (Weithmann et al. 2018). More than 700 kt of MPs have been estimated to be transferred annually to agricultural lands in Europe and North America, originated from urban sewage sludges used as farm manure, adversely affecting soil ecosystems, crops and livestock either directly or via the presence of other toxic substances embedded in them (Nizzetto et al. 2016).

Inappropriate or uncontrolled recycling (mostly implemented in developed countries) is also one of the major release sources of SoC present in plastics, such as brominated flame retardants (BFRs), polybrominated biphenyl ethers (PBDEs), toxic metals, etc., that contaminate lands (Mousa et al. 2015; Song and Li 2014; Tue et al. 2016). Open burning or uncontrolled incineration of plastics, also occurring in many of these countries, is another major source of contamination for both soil and air. Actions like the aforementioned ones release carbon dioxide (CO_2_) and black carbon (Wiedinmyer et al. 2014); burning plastics that contain BFRs, as well as several additives (Hahladakis et al. 2018b), is a significant source of air pollution, including the emission of unintended POPs, e.g. chlorinated and brominated dioxins, furans and polychlorinated biphenyls (PCBs) (Hahladakis et al. 2018b; Verma et al. 2016); it further poses several threats to all living organisms, since toxic particulate matter could potentially settle on crops or in waterways, degrading water quality and entering the food chain.

MPs may also act as contaminants that could potentially degrade water quality and, consequently, affect its availability, thereby harming the fauna (Wagner et al. 2014).

### What are the solutions for plastic litter prevention? Future recommendations

Plastics production, consumption, recovery and recycle are a nexus affected by a web of different facets, occurring at different parts of the supply chain. Therefore, a multidimensional appraisal of the system as a whole and several changes/interventions are needed to be carried out in order to prevent leakages to the environment and for sustainable developments to occur (Iacovidou et al. 2019).

To begin with, it seems that there is a lack of legislation, especially in underdeveloped or developing countries, to control the contamination of water bodies and ecosystems (including surface waters, the benthos and sediments) from plastics (mostly MPs) (Barnes et al. 2009; Tibbetts 2015). Governments should cooperate not only on local but also on global level to regulate the main origins and sources of MPs. Taking into consideration that this issue is a relatively new one and of increasing concern, additional resources should be allocated to further research on the long-term effects and consequences that plastics, and additives contained in them, have on living organisms (Hahladakis et al. 2018b; Oehlmann et al. 2009). Filling this knowledge gap could potentially contribute to the lack of certain regulations, regarding, e.g. the prevention or limitation in the use of bisphenol A (BPA).

Apart from clear and strict legislation, technological advancements in the area of plastics collection from all media (water and soil) should be implemented. In the past, manta trawlers (a hybrid like between a fish trawler and a plankton tow) were used for collecting buoyant plastics (Ryan et al. 2009). Nonetheless, they were deemed insufficient for oceanic application and were later substituted by autonomous devices (“drones”) that can tow a trapping net, thus removing more efficiently plastic debris (Boyle 2012). The use of sonic transmitters is also encouraged to prevent marine organisms from getting trapped in the nets (Sigler 2014).

Another innovative technique that uses ellipsoid bodies is capable of detecting coloured plastic debris, mostly on shorelines and beaches, using webcam taken photos. This technique generates colour references, via the use of a uniform colour space, to detect plastic pixels and is also able of removing any mistakenly detected pixels by the application of a composite image method (Kataoka et al. 2012). In addition, the webcam monitoring of plastics in the beaches and shorelines is a valuable functionality of this method since it enables a remote measuring of the level of contamination, thereby facilitating any potential planning of systematic clean-up initiatives (Kataoka et al. 2012).

Furthermore, incentivizing and educating the public on the seriousness of the situation caused by plastic litter are considered an absolute necessity in stepping towards shifting people’s behaviour with regard to plastic consumption, use and disposal habits. It is a priority issue that should be placed on the top of the international political agenda. Although dumping of plastic waste is forbidden by the International Convention for the Prevention of Pollution from Ships (MARPOL) Annex V, many people are unaware or tend to ignore this.

Several campaigns organized by relevant policy makers, marine businessmen, industries and stakeholders should take place, so as further light is shed on the urgency of this international matter. Worldwide examples of inspirational actions include the “Clean up Kenya”, the “Bye-Bye Plastic Bag” and the “Last Straw”. In addition, San Francisco has banned plastic bags and bottles (Levin 2017). It is also noteworthy that the Indian state of Maharashtra and the EU are banning single-use plastics. Informing and incentivizing the public into participating in individual actions would be an essential help in addressing the global plastic litter issue. Changing our own waste-disposing mentality and attitude could, in fact, be the most promising solution. With time it will, eventually, reduce the total amount of inappropriately disposed plastic waste.

Finally, plastic industries should be responsible for the end-of-life (EoL) of their products, using, as possible, biodegradable material that will be more easily degraded by microorganisms (such as bacteria and fungi), thereby reducing even more the lifetime of these bioplastics, when/if entering the marine environment (Gregory and Andrady 2003).

## Conclusions

Plastic waste is a global issue of increasing concern, and actions need to be taken in all stages of their lifecycle, design stage, manufacturing/production stage and disposal/recycling stage. These are necessary requirements should we wish to make it possible for plastics to circulate back into technical and biological cycles and maximize their value.

Although inappropriate disposal of plastic waste is legislatively prevented by the International Convention for the Prevention of Pollution from Ships (MARPOL) Annex V, most people are unaware or tend to ignore this. To further underpin the importance and urgency of this issue, various campaigns should be organized by the relevant policy makers, marine businessmen, industries and stakeholders, so as further shed light on this international matter. Large multinational organizations, such as the United Nations Environment Programme (UNEP) and the International Maritime Organization (IMO), should also contribute to this, and individually, on a global scale.

Plastic industries should strive in increasing the plastic waste recycling rates, adopting the “design for recycling” concept and taking full responsibility of their EoL products; this necessitates a transparent communication and collaboration between all stakeholders’ involved in the plastic value chain. It requires effort from those producing and specifying the plastic products used, through to collectors, sorters and reprocessors.

It is a priority issue that should be placed on the top of the international political agenda. People should strive forward to resolve any problems reasonably and peacefully, maintaining at the same time and above all earth’s environmental balance and human welfare. This is a legacy that we owe to pass on.

## Abbreviations

approx.: Approximately
BFRs: Brominated flame retardants
BPA: Bisphenol A
ca.: Circa (Latin term for “approximately” or “about”)
CE: Circular economy
EC: European Commission
EEE: Electrical and electronic equipment
EoL: End-of-life
HDPE: High-density polyethylene
IMO: International Maritime Organization
LDPE: Low-density polyethylene
MPs: Microplastics
PBDEs: Polybrominated biphenyl ethers
PCBs: Polychlorinated biphenyls
PET: Polyethylene terephthalate
POPs: Persistent organic pollutants
PP: Polypropylene
PS: Polystyrene
PVC: Polyvinyl chloride
SoC: Substances of concern
UNEP: United Nations Environment Programme
